# Ameliorative effects of vitamins-loaded flavoured nanophytosomes fortified with star anise volatile oil against CsA-Induced liver and kidney injury in rats: Application in functional ice cream

**DOI:** 10.1016/j.heliyon.2023.e23894

**Published:** 2023-12-19

**Authors:** Manal M. Ramadan, Rasha S. Mohamed, Amal G. Hussien, Ola A.M. Mohawed, Ahmed M. Mabrouk, Abeer E. Mahmoud, Kadry Z. Ghanem, Tamer M. El-Messery

**Affiliations:** a Chemistry of Flavour and Aroma Department, National Research Centre, Cairo, Egypt; b Nutrition and Food Sciences Department, National Research Centre, Cairo, Egypt; c Biochemistry Department, Biotechnology Research Institute, National Research Centre. Cairo, Egypt; d Hormones Department, Medical Research and Clinical Studies Institute, National Research Centre. Cairo, Egypt; e Dairy Science Department, National Research Centre, Cairo, Egypt; f International Research Centre “Biotechnologies of the Third Millennium”, ITMO University, St. Petersburg, 191002, Russia

**Keywords:** Flavoured nanophytosome, Vitamins, Immune system, Star anise, Ice cream, Phytosome encapsulation

## Abstract

This study investigated the effect of flavoured nanophytosomes loaded with vitamins A, E, D, B complex, folic acid, and C, as well as zinc on the immunosuppressive cyclosporin A (CsA)-induced liver and kidney injury in male rats. The vitamins flavoured nanophytosomes (VFnPs) were characterized in terms of particle size, zeta potential, encapsulation efficiency. Ice cream was flavoured with star anise volatile oil to mask the VFnPs' flavour and unacceptable taste. The study found that treatment with CsA alone resulted in increased (P > 0.05) levels of creatinine, urea, and MDA, as well as the activities of AST and ALT, while the levels of SOD, CAT, GST, proteins, CD4, INF-ᵧ, IL-6, IL-1β, and TLR4 decreased (P > 0.05). However, the group that received CsA simultaneously with VFnPs showed a significant (P > 0.05) decrease in the levels of creatinine, urea, and MDA, as well as the activities of AST and ALT, and increased (P > 0.05) levels of SOD, CAT, GST, proteins, CD4, INF-ᵧ, IL-6, IL-1β, and TLR4. The increase in the ratio of VFnPs had little effect on the physiochemical and sensory evaluation of the ice cream. Finally, the study suggests that VFnPs could potentially protect against CsA-induced liver and kidney injury and serve as a promising natural therapy for treating such conditions.

## 1 Introduction

Cyclosporine A (CsA) is the immunosuppressor most frequently used in transplant surgery and in the treatment of autoimmune diseases because of its specific inhibiting effect on the signal transduction pathways of cell T receptor. CsA-induced kidney and liver damage is the main clinical problem associated with CsA therapy in which oxidative stress (OS) is the conceivable accountable mechanism. Therefore, the use of antioxidants is a useful tool to reduce CsA adverse effects [1,2]. Vitamins can protect our body against a lot of diseases, like beriberi, night blindness and scurvy. Most countries allow food additives with vitamins extracted from plants as an optimal intake of vitamins [3]. Micronutrient supplementation guidelines were disseminated by the Food and Agriculture Organization (FAO) and the World Health Organization (WHO) to provide the best approach for plant additives [4]. To fully exploit the vitamins in humans, phytosome technique was used. Phytosome is one of the novel lipid-based vesicular delivery systems used in the formulation of botanical-based nutraceuticals [5] and medicines [6,7]. Phytosome can improve bioavailability and absorption in the gastrointestinal tract for bioactive compounds extracted from herbs, such as vitamins and minerals [8]. Phytosomes also enhance antimicrobial and antioxidant activities of nutraceutical compounds and protect bioactive compounds during food processing, heat treatment (sterilization & pasteurization) and storage [9]. The interaction between phosphatidylcholine molecules and micronutrients via hydrogen bonds increases the stability of phytosomes and forms a water and lipid soluble complex [10]. However, the phytosome technique is underresearched as a novel food delivery system. During food manufacturing and storage there are main disadvantages such as insolubility in aqueous phase, strong odor, biodegradation, alkaline conditions, and sensitivity to heat. These were reasons for using a novel method like phytosome encapsulation [11].

The innate and adaptive immune systems are the two main immune system components. The effectiveness of a person's immune system is significantly influenced by the nutritional status of these systems. The body's capacity to maintain innate immune responses may be compromised by under-nutrition because of insufficient consumption of micronutrients [12]. Nutritional approaches to support immune system function are frequently overlooked in public health talks on immunity and illnesses, although nutrition is key in immune function. Numerous vitamins, such as vitamins A, B6, B12, C, D, and E, as well as trace minerals, such as zinc, iron, selenium, magnesium, and copper, promote the innate and adaptive immune systems. Immune function is negatively impacted by micronutrient deficiencies or inadequate status, which might lower resistance to infections [13]. Micronutrients' molecular functions in immune system have lately received extensive description. Most micronutrients play pleiotropic actions in promoting immunological health. Regarding innate immunity, the vitamins and minerals enhance the proliferation, differentiation, and motility/chemotaxis of innate cells, as well as the phagocytic and killing (e.g., oxidative burst) activities of neutrophils and macrophages, the induction of inflammation and its recovery (e.g., cytokine production and antioxidant activity). Additionally, they promote adaptive immunity through the development of memory cells, cytokine synthesis, antibody formation, lymphocyte differentiation, proliferation, and homing [14].

In addition to being utilized in modern medicine in East Asia, star anise (*Illicium verum* Hook f) is regarded as a key species in Traditional Chinese Medicine. The presence of beneficial secondary metabolites like sesquiterpenoids, monoterpenoids, phenylpropanoids, sesquilignans, shikimic acid, seco-prezizaane-type, sesquiterpenoids, and flavonoids determines the biological activity of star anise. Recent pharmacological research on star anise essential oil has validated its antioxidant, antibacterial, and antifungal properties [15,16].

Ice cream is a delicious and popular frozen dairy product appreciated by a very broad spectrum of consumers, made from milk and/or milk products combined with other ingredients such as sweeteners, emulsifiers, stabilizers and flavouring agents [17]. Frozen functional dairy products are very important vehicles of bioactive ingredients for enhancing nutritional health benefits [18]**.** There are different types of ice cream with new functional ingredients that are available on the markets worldwide. In addition, numerous ice cream formulations with functional properties have been developed, for example, ice cream with natural antioxidants [19]**,** with dietary fibers and probiotics [20]**,** with polyunsaturated fatty acids [21]**,** and with vitamins and minerals [22]**.**

This study aims to investigate the effect of flavoured nanophytosomes loaded with vitamins on the immunosuppressive CsA-induced liver and kidney injury in rats and to develop ice cream as a functional food fortified with vitamins-loaded flavoured nanophytosomes (to protect the bioactive vitamines during food processing) as an antioxidant and an anti-inflammatory agent against CsA-induced liver and kidney injury, and to flavour the final product with star anise oil to overcome the major problems of off-flavour and unacceptable taste.

## 2 Materials and methods

### 2.1 Materials

Star anise (*Illicium verum*) was procured from a specialized aromatic plant market in Cairo – Egypt. Vitamins (A, E, D, B complex, Folic acid and C) and element (Zn) were procured from local pharmacy in Cairo – Egypt. Soy lecithin granules were obtained from Solgar, Inc. (Leonia, NJ 07605, USA). All chemicals and solvents for HPLC and GC mass were from Sigma-Aldrich (Sternheim, Germany).

### 2.2 Isolation of star anise essential oil

Hydro-distillation method was used to isolate the star anise essential oil in the Clevenger's apparatus for 3 h [23]**.**

### 2.3 Determination of total phenolic content and antioxidant activity

Total phenolic content and reducing power assessment DPPH radical scavenging activity were determined according to **Zhang et al.,** [24] and **Bose and Kim** [25]**,** respectively.

### 2.4 Gas chromatography-mass spectrometry (GC – MS) analysis

The volatile compounds were determined using gas chromatography coupled with mass spectrometry. The instrument used was a Hewlett–Packard model 5890 GC coupled with a Hewlett–Packard-MS (5970) MS. The analysis was performed using a DB-5 column with dimensions of 60 m × 0.32 mm i.d. × 0.25 μm film thickness. The temperature program for the oven started at 50 °C for 5 min and then increased from 45 to 250 °C at a rate of 5 °C/min. The flow rate of helium gas was 1.1 ml/min. The sample size used was 2 μl with a split ratio of 1:10, and the injector temperature was set at 220 °C. Mass spectra were obtained in the electron impact mode (EI) at 70 eV, and the scan *m*/*z* range was from 39 to 400 amu. The identification of isolated peaks was achieved through matching with data from the mass spectra library (National Institute of Standard and Technology, NIST), comparison with published data, and utilizing authentic compounds [26].

### 2.5 Preparation of flavoured vitamins-loaded nanophytosomes

Nanophytosomes technique (thin layer hydration) was used to encapsulate vitamins (A, E, D, B complex, Folic acid and C), element (Zn) and their mixture according to **Nazari et al.** [11], with some modification. The ratio of soy lecithin and vitamins was 1:1. 60 mg. Vitamins and same amount of soy lecithin were dissolved in absolute ethanol and added to a round flask of 100 ml, then evaporated at 60 °C/60 min by a vacuum rotary evaporator (Heidolph, Laborota 4002 control, Germany) to remove all ethanol and form a thin dry film at the round bottom of the flask. Then, 10 ml of distilled water (60 °C) was added in a round flask to hydrate the thin dry film, then star anise oil (3 %) was added and homogenized using probe sonication (Hielscher – 130 W, 20 kHz, Germany) (15 min cycles with 30 s rest between cycles). The size of phytosomes was decreasing.

### 2.6 Characterization of vitamins-loaded flavoured nanophytosomes

#### 2.6.1 Particle size and zeta potential

The particle size and zeta potential of VFnPs were measured using the Malvern Zetasizer Nano Z, (Worcestershire, UK)

#### 2.6.2 Transmission electron Microscopy (TEM)

The morphology of nanophytosomes was studied and visualized using transmission electron microscopy (TEM) (JEM-2100 Electron Microscope Instruments, China) after dry sample coating with gold (DST3, Nanostructured Coating Co., Tehran, Iran).

#### 2.6.3 Loading parameters

Encapsulation efficacy (EE) of VFnPs was calculated using the centrifugation method [27]. An amount of nanophytosomes was centrifuged (3 k-30, Sigma, Germany) at 15000 rpm/15 min/4 °C. The free vitamins were determined by HPLC and the following equations were used to calculate EE:

(EE)%=(TotalVitamins‐Freevitamins)/(Totalvitamins)×100

#### 2.6.4 HPLC conditions

The column Agilent C18 (100 mm i.d., 3.5 μm × 4.6 mm) was used to determine free vitamin. The methanol: acetonitrile (65:35) was used as a mobile phase and the flow rate was 1 ml/min 20 μl of the sample solutions was injected. The DAD was adjusted to 295 nm. The column temperature was 40 °C.

### 2.7 Biological study

#### 2.7.1 Animals

We obtained adult male Wistar rats from the Animal Care Unit of the National Research Centre in Egypt that were 120 days old and weighed between 150 and 200 g. Animals were kept in plastic transparent cages in the animal house with lights on between 7:00 a.m. and 7:00 p.m., a room temperature of 22± 2 °C, and a humidity level of 50 ± 10 %. Food and water were freely available to the rats.

#### 2.7.2 Animals' diet

AIN-93 balanced diet: 58.5 % maize starch, 5 % fibre, 3.5 %, 10 % corn oil, 10 % sucrose, 12 % casein-supplemented protein was mixed and contained 1 % AIN-93 vitamins and AIN-93 salt according to **Reeves et al.** [28]**.**

### 2.8 Evaluation of the flavoured vitamins-loaded nanophytosomes for improving immunity and protecting the liver against cyclosporine-A

The animals were kept under observation for about 7 days before starting the experiment for acclimatization. Then, the animals were classified into 3 groups (N = 6/group) according to the treatment schedule. The control normal group received orally 1 ml saline (vehicle), immunosuppressive group received cyclosporine-A (CsA) dissolved in saline orally in a dose of 15 mg/kg body weight daily for 8 weeks [29]**.** The third group received CsA simultaneously with the VFnPs (3 mg of the vitamins’ nanophytosomes) daily for 8 weeks. The Ethical Committee for Medical Research of the National Research Centre in Egypt approved the experimental protocol (Code No. 19182.)

At the end of the trial period (8 weeks), Diethyl ether was used to anaesthetize the animals. After that, blood was drawn from the retro-orbital venous plexus and placed into EDTA-free tubes to separate the sera, which were then chilled to −80 °C for further analysis. The animals were then killed via cervical dislocation, and the liver was removed right away.

### 2.9 Biochemical analyses

According to **Raeeszadeh et al.** [30], the ferric reducing antioxidant power (FRAP) method, was used to determine plasma total antioxidant capacity (TAC). This method measured the plasma's capacity to lower ferric ions. The FeIII-TPTZ combination has the maximum optical absorption at 593 nm at an acidic pH when it is reduced to FeII and produces a blue color.

Colorometry and using UVPC spectrophotometer (Jasco V-730, serial No. A 112361798, Japan), serum total protein, the activities of aspartate transaminase (AST), and alanine transaminase (ALT), creatinine, urea, and albumin, respectively were determined according to the methods described by **Rheinhold & Seligron** [31]**, Reitman & Frankel** [32] **Larsen** [33]**, Fawcett and Scott** [34]**, and Doumas et al.** [35]**.**

Liver homogenates (10 % w/v) in a cold homogenization buffer (100 mM potassium phosphate buffer, pH 7.4) were prepared and centrifuged, then the supernatants were analyzed for tumor necrosis factor-alpha (TNF-α), malondialdehyde (MDA), glutathione S. transferase (GST), catalase (CAT), and superoxide dismutase (SOD) activity. Liver TNF-α was determined using an Eliza kit (Cat N.: E-EL-R2856) (Elabscience Biotechnology Co., Ltd, Wuhan, China). MDA was determined using thiobarbituric acid (TBA) method. Briefly, 1 ml trichloroacetic acid (25 mmol/L) were added to 200 μl of the supernatants, mixed for 1 min before being submerging in a bath of boiling water. Then, a measurement of the pink solution's absorbance at 532 nm was carried out. After preparing various MDA concentrations in N-butanol (0.2 μM–2 μM), a standard curve was prepared [36]**.** The measurment of GST activity was based on the conjugation of 1-chloro-2,4-dinitrobenzene (CDNB) with reduced glutathione. At 340 nm, absorbance increases in parallel with conjugation [37]**.** According to **Beers and Sizer** [38], the spectrophotometric measurement of H_2_O_2_ degradation, which was directly correlated with the decrease in its absorbance at 230 nm per time unit, used as the basis for an estimation of CAT activity. The role of SOD in converting the damaging radical O2-superoxide to molecular oxygen and hydrogen peroxide during oxidative processes was used as the basis to assess its activity [39].

Serum cluster of differentiation 4 (CD4), cluster of differentiation 8 (CD8), interferon-gamma (INF-γ), interleukin 6 (IL-6), interleukin 1-beta (IL-1β) and toll-like receptor 4 (TLR 4) were determined using rats CD4 (Cat. N.: E-EL-R2459), CD8 (Cat N.: E-EL-R0219), INF-γ (Cat N.: E-EL-R0009), IL-6 (Cat N.: E-EL-R0015), IL-1β (Cat N.: E-EL-R0012) and TLR 4 (Cat N.: E-EL-R0990) enzyme-linked immunosorbent assay kits (Elabscience Biotechnology Co., Ltd, Wuhan, China).

### 2.10 Ice cream formulation

The ice cream mixes (2 Kg each as shown in Table 1) were formulated to contain 12 % fat, 14 % SNF, stabilizers 0.5 % and 16 % sugar. The ingredients of all treatments were homogenized well and heated at 90 ± 1 °C/5 min [40]. The mixtures were cooled to 5 °C. Then, the mixtures were divided into three parts: the first one as a control (C). The second one (T1) was formed by adding 0.166g of VFnPs. The third one (T2) was formed by adding 0.330g of VFnPs (This concentration according to daily intake from vitamins). The ice cream mixes were prepared according to **Mabrouk et al** [18]**.**

**Table (1):** Formula of ice cream mixes (g/2 kg).

Ingredients	Control	T1	T2
**Sugar**	320	320	320
**SMP**	200	150	100
**Fresh milk**	1270	1270	1270
**Fresh cream**	200	200	200
**CMC**	10	10	10
**MP + SA**	–	50	100
**VFnPs**	–	0.166	0.330
**Total (g)**	2000	2000	2000

- **SMP**= Skim milk powder – **CMC**= Carboxymethyl cellulose.

**MP** = milk powder- **SA** = star anise. **VFnPs** = vitamins-loaded flavoured nanophytosomes.

### 2.11 Physiochemical analysis of ice cream

#### 2.11.1 Specific gravity and weight per gallon

Specific gravity and weight per gallon of mixes and the ice cream were calculated according to **Sadek *et al.*** [41] **and Arbukle *et al*** [40]**,** respectively**.**

#### 2.11.2 pH values, titratable acidity and ash contents%

The pH values, titratable acidity and ash contents of ice cream samples were measured according to **Mabrouk *et al.*** [18]**, AOAC** [42]**,** respectively.

#### 2.11.3 Overrun and melting rate

The overrun and melting dawn of ice cream was determined according to **Akın *et al.*,** [43].

#### 2.11.4 Sensory evaluation of ice cream

Ice cream samples were sensory evaluated after 24 h hardening at 20 °C by ten well-trained panellists. The samples were scores for flavour (45), body &texture (35), colour (10) and melting quality (10) as suggested by **Arbukle *et al* (986).**

### 2.12 Statistical analysis

For statistical analysis SPSS (20) was used., Using ANOVA one-way the data were statistically evaluated, and the mean standard error was reported, then the Duncan test followed. The statistical significance of the difference utilizing probability is P ≤ 0.05.

## 3 Results and discussion

### 3.1 The total phenolic content and antioxidant activity of star anise oil

Results shown that the amount of total phenolic content was 265.01 ± 1.50 μg GAE gallic acid equivalent/ml. Anise oil showed good reducing power (0.188 ± 0.01 mg Vit C equivalent/ml). The reducing power of essential oil might be due to their hydrogen donating ability. The components present in the oil could act as good reductant. The total phenolic content of star anise oil showed positive correlation with its reducing capacity [44]. Star anise oil has strong scavenging activity towards DPPH radical (89.72 % ± 2.03). Our results agreed with **Singh et., al.** [45]**,** who reported that the method is based on the reduction of DPPH radicals and formation of non-radical form (DPPH-H), and that star anise oil is able to perform this reaction and is considered as radical scavenger and hence antioxidant. Star anise oil showed the strongest scavenging power that was even higher than BHA and BHT [45,46]**.**

**Fig. (1):**
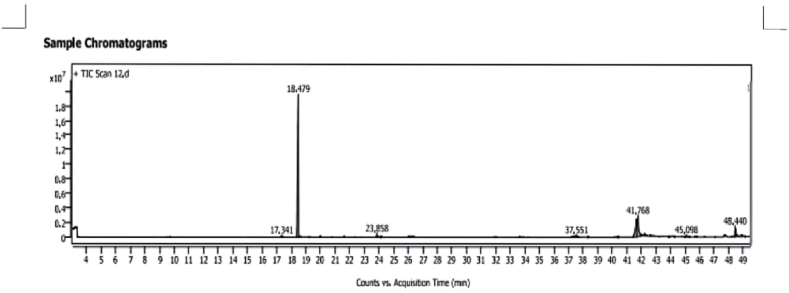
GC-MS Chart of star anise volatile oil.

Figure (1) Illustrates the GC-MS chromatogram of Egyptian star anise extracted by hydro-distillation and the nine identified compounds listed in **Table (2)** represent about 97.6 % of the total detected compounds. The major compounds were anethole (63.47 %), oleic acid (13.72 %) and linoleic acid (11.99 %). The main compounds in our results matched with results of the Indian and Italian star anise oil [47,48]**.**

### 3.2 Characterization of nanophytosomes

#### 3.2.1 Particle size and zeta potential

In this study, the particle size (nm), PdI, zeta -potential (mV) and encapsulation efficiency (EE) of VFnPs are shown in Table 3. According to these results, a variation was found between particle size for vitamins (A, D, E, C, F, B), Zn and Mixture loaded nanophytosomes were 213.6, 81.36, 228.5, 214.8, 107.3, 168.4167.4, and 149.4, respectively. These results clearly indicate that the vitamins maximized the size of the VFnPs. **Gibis et al.,** [49] stated that the size of the phytosomes was dependent on the quality of the materials loaded in the phytosomes, which explains the present finding. Phytosomes agglomerated into nano-size micelles (approximately 100–300 nm) [50].

**Table 2:** Volatile compounds of star anise essential oil using GC-MS.

Peak No.	Components	Area %	Retention time (RT) (min)
1	Anisaldehyde	0.69	17.341
2	Anethole	63.47	18.479
3	Ethyl Vanillin	1.02	23.858
4	Palmitic acid	2.48	37.551
5	Linoleic acid	11.99	41.648
6	Oleic Acid	13.72	41.768
7	Glycidyl palmitate	1.24	42.226
8	β-Monolinolein	0.66	45.098
9	Glycidol oleate	2.33	48.44

**Table 3:** Encapsulation efficiency, Particle size and zeta potential of flavoured vitamins –loaded phytosomes.

flavoured vitamins –loaded phytosomes	Encapsulation efficiency	Particle size (nm)	PdI	Zeta Potential (mV)
**A**	89.96^a^±1.04	213.6^e^±2.645	0.358^d^ ± 0.021	−11.2^a^±0.141
**D**	89.23^a^±2.65	81.36^d^ ± 2.178	0.375^b^ ± 0.023	−11.9^a^±0.495
**E**	90.00^a^±3.45	228.5^c^±2.503	0.423^a^±0.016	−17.7^bc^±0.071
**C**	89.98^a^±1.05	214.8^b^ ± 1.662	0.348^c^±0.022	−32.1^b^ ± 0.240
**F**	89.05^a^±2.95	107.3^b^ ± 1.251	0.354^c^±0.019	−22.0^b^ ± 0.240
**B**	90.01^a^±1.58	168.4^a^±2.017	0.460^e^±0.055	−18.1^c^±0.212
**Zn**	86.80^a^±2.45	167.4^a^±3.917	0.351^e^±0.023	−29.7^c^±0.125
**Mix**	–	149.4^a^±1.258	0.344^e^±0.064	−34.3^c^±1.253

The same letter in a column indicates a non-significant difference, whereas a different letter, with 0.05 probability, indicates a significant difference. In terms of mean values and standard error, the data are expressed.

The particle distribution index (PDI) defines the particle distribution curve. The obtained results indicated almost the same PDI for different percentagesof the loaded vitamins except for the largest one (vitamin B), which showed narrower size distribution of the formed phytosomes.

The zeta-potential values of the VFnPs ranged from - 11.2 to - 34.3 mV. This might be because vitamins (positive charge) interacted with the phosphate group in phosphatidylcholine (the negatively charged) giving the VFnPs (negative charge). In general, zeta-potential values of above −30mV or under +30 mV were observed to protect particles and minimize electrostatic and congregating force between nano-phytosomes, which might create large collection of nanophytosome nanovesicules [51].

#### 3.2.2 Encapsulation efficiency (EE)

Encapsulation efficiency (EE) was determined via calculation of free vitamins content in VFnPs. A smaller amount of free vitamins content means high encapsulation efficiency. There was no significant (p < 0.05) difference between EE of VFnPs. To assess the effectiveness of nanocarriers in delivering active substances, EE is a critical factor in nano-based delivery systems [52]. The achieved results of VFnPs, that were prepared via three different formulations and showed the exceptional capacity of nano-phytosomes as carriers, are shown in Table 3. **Nazari et al.,** [11] investigated the garlic essential oil (GEO) loaded in nano-phytosomes as a new phytochemicals delivery vehicles using three varied techniques, and found that GEO nano-phytosomes using homogenization-probe sonication (the same technique used in this study) is promising with a size of 115 nm and EE of 86.00 % (which is similar to the values found for the vitamins loaded nano-phytosomes presented in this study).

#### 3.2.3 Transmission electron microscopy (TEM)

**Fig. (2):**
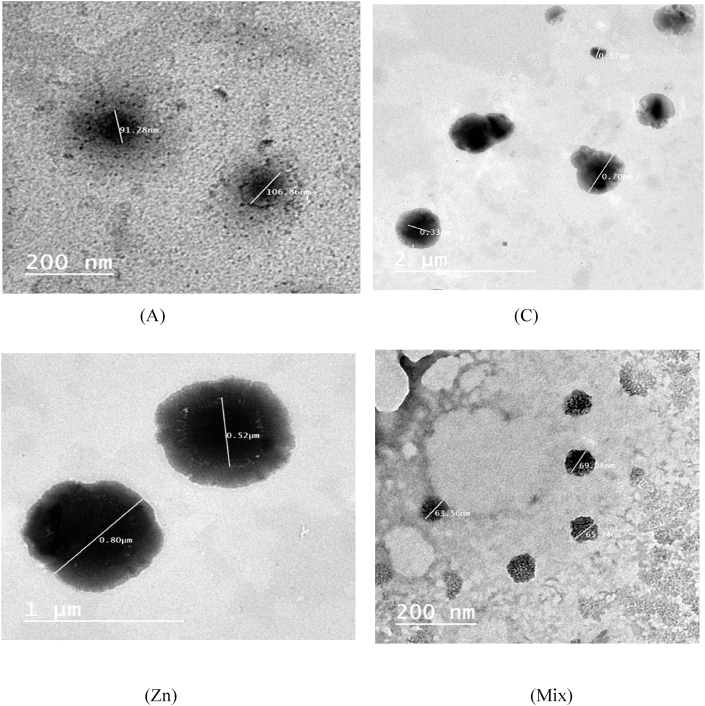
TEM micrograph of flavoured vitamins (A and C), Zn and Mixture loaded phytosomes with obtained at a scale of 200–2000 nm.

Transmission electron microscopy (TEM) was used to study the morphology of the VFnPs, which revealed spherical vesicles in all pictures (Fig. 2). Moreover, the particles size distribution was existence of certain aggregates, indicating was homogemized, which is consistent with the PDI values reported (Table 3). The size for particle via image is agree with particle size which measured using Malvern Zetasizer that have reported a size distribution between 100 and 300 nm for phytosomes used in the encapsulatation [53,54].

### 3.3 Role of the vitamins-loaded flavoured nanophytosomes in improving immunity, antioxdant and protecting the liver and kidney against cyclosporine-A

According to Table 4, the control positive group reported considerably (P > 0.05) higher values for AST, ALT, creatinine, and urea than the normal control group. Compared to the normal control group, the results for total protein and albumin dramatically (P > 0.05) fell in the control positive group. Rats given the VFnPs had lower (P > 0.05) levels of AST, ALT, creatinine, and urea than the control positive group. Rats given the VFnPs had greater (P > 0.05) total protein and albumin values than the control group of positive animals.

**Table 4:** Liver and kidney functions of different experimental groups.

	AST (U/l)	ALT (U/l)	T. protein (g/dl)	Albumin (g/dl)	Creatinine (mg/dl)	Urea (mg/dl)
**Control negative**	32.78^a^±0.76	21.55^a^±0.82	7.31^b^ ± 0.14	4.28^b^ ± 0.11	1.40^a^±0.05	27.55^a^±0.31
**Control positive**	64.21^b^ ± 1.10	38.37^c^±0.57	4.13^a^±0.06	2.34^a^±0.12	1.89^b^ ± 0.05	32.41^b^ ± 0.84
**flavoured vitamins- loaded nanophytosomes**	34.41^a^±0.45	25.51^b^ ± 0.22	7.07^b^ ± 0.09	4.12^b^ ± 0.08	1.51^a^±0.04	27.94^a^±0.30

The same letter in a column indicates a non-significant difference, whereas a different letter, with 0.05 probability, indicates a significant difference. In terms of mean values and standard error, the data are expressed.

Table 5 shown that the total antioxidant, liver GST, liver SOD, and liver catalase values were considerably (P > 0.05) lower in the control positive group than in the normal control group. As opposed to the normal control group, the control positive group's liver MDA and TNF- levels considerably (P > 0.05) rose. The liver GST, SOD, and catalase values in VFnPs-treated rats were higher (P > 0.05) than those in the control positive group. Rats given the vitamin-loaded nanophytosomes had considerably (P > 0.05) lower liver MDA and TNF levels than the control-positive group.

**Table 5:** Oxidative markers of different experimental groups.

	MDA (nmol/g)	T. antioxidant (mM/l)	GST (U/g)	SOD (U/g)	Catalase (μmol/g)	TNF-α (pg/g)
**Control negative**	16.80^a^±0.56	2.07^b^ ± 0.07	28.18^c^±0.42	4.85^c^±0.12	443.67^c^±3.85	15.29^a^±0.41
**Control positive**	33.00^c^±0.86	1.04^a^±0.04	15.84^a^±0.50	2.64^a^±0.17	241.50^a^±2.95	31.61^c^±0.49
**Flavoured vitamins- loaded nanophytosomes**	23.47^b^ ± 0.86	1.76^b^ ± 0.18	23.42^b^ ± 0.38	3.55^b^ ± 0.13	406.17^b^ ± 2.77	17.37^b^ ± 0.36

The same letter in a column indicates a non-significant difference, whereas a different letter, with 0.05 probability, indicates a significant difference. In terms of mean values and standard error, the data are expressed.

According to Table 6, the control positive group recorded considerably (P > 0.05) higher CD8, INF-ᵧ, IL-6, IL-1β, and TLR4 values than the normal control group. As opposed to the normal control group, the CD4 value in the control positive group considerably (P > 0.05) reduced. Rats given the VFnPs had lower levels of CD4, INF- ᵧ, IL-6, IL-1 β, and TLR4 than the positive control group. Rats given the VFnPs had considerably (P > 0.05) greater CD8 values than the control-positive group.

**Table 6:** Immunity markers of different experimental groups.

	CD4 (pg/ml)	CD8 (pg/ml)	INF-ᵧ (pg/ml)	IL-6 (pg/ml)	IL-1β (pg/ml)	TLR4 (pg/ml)
**Control negative**	702.67^c^±2.40	332.50^a^±3.77	29.64^a^±0.73	78.48^a^±0.72	10.89^a^±0.29	0.33^a^±0.01
**Control positive**	520.33^a^±2.97	497.50^b^ ± 3.06	40.35^b^ ± 0.85	164.65^b^ ± 1.24	34.05^c^±0.35	0.90^c^±0.01
**Flavoured vitamins- loaded nanophytosomes**	693.50^b^ ± 2.93	336.83^a^±3.25	30.67^a^±0.67	80.66^a^±0.73	12.68^b^ ± 0.77	0.43^b^ ± 0.01

The same letter in a column indicates a non-significant difference, whereas a different letter, with 0.05 probability, indicates a significant difference. In terms of mean values and standard error, the data are expressed.

The liver plays a key role in the metabolism of drugs, foods, and many compounds, so changing its function endangers a person's health. Liver damage could eventually lead to the increased serum activities of some liver enzymes Although ALP, GGT, and LDH are found in many tissues, some previous studies have shown that the most common reason for these enzymes' increase is a defect in liver function [55].

The findings of the current investigation showed that cyclosporine-A caused harm to the liver and kidneys of rats, as evidenced by an increase in ALT, AST, creatinine, and urea and a decrease in total protein and albumin levels in the serum. Reduced total antioxidant levels, elevated levels of lipid peroxidation product (MDA), and decreased SOD, catalase, and GST activity were indicators of cyclosporine-A-induced oxidative stress. The immunosuppressive and inflammatory effects of cyclosporine-A were also demonstrated by changes in the levels of CD4, CD8, INF-ᵧ, IL-6, IL-1β, TLR4, and hepatic TNF-α. When cyclosporine-A causes liver damage, oxidative stress is a major factor. Several cells are stimulated to undergo oxidative stress, apoptosis, and autophagy as a result of cyclosporine-A therapy, which produces ROS, an important factor [56]. Cyclosporine-A stimulates intra-mitochondrial Ca^2+^, inhibits mitochondrial glucose metabolism and ATP synthesis, and increases ROS, which causes lipid peroxidation and elevates its byproducts such MDA [57]. The important antioxidant GSH, which helps keep mitochondria and cell membranes healthy by converting lipid peroxides into harmless byproducts, is similarly decreased by cyclosporine-A administration. Additionally, the antioxidant enzyme activity of GST, SOD, reduced glutathione, glutathione peroxidase, and glutathione reductase are decreased by cyclosporine-A [58]. To regulate the effects of cyclosporine-A, ROS elimination is therefore regarded as a crucial target.

The current study's findings suggested that the prepared vitamins-loaded nanophytosomes prevented liver and kidney damage, as seen by rising levels of total protein and albumin and declining levels of ALT, AST, creatinine, and urea. Additionally, the vitamins-loaded nanophytosomes reduced hepatic levels of lipid peroxidation product (MDA), boosted SOD, catalase, and GST activities, and decreased hepatic levels of MDA as a result of oxidative stress. Increased serum CD4 levels, decreased serum CD8 levels, INF-ᵧ, IL-6, IL-1β, TLR4, and hepatic TNF-α all indicated immunosuppression and inflammation, which were both inhibited by the prepared vitamins-loaded nanophytosomes. The benefit of prepared vitamins-loaded nanophytosomes in cyclosporine-related effects prevention may be attributed to the increased consumption of vitamins (A, B complex, C, D, E and folic acid) and zinc which were encapsulated into nanophytosomes.

Vitamin A, commonly known as retinoic acid, is a member of the retinyl-ester family and regulates several genes involved in both innate and adaptive immune responses. Adaptive and innate immunity are supported by vitamin A role as T-cell effectors [59]. Retinoid directly stimulates the expression of Interferon stimulated genes (ISGs), including retinoic acid-inducible gene I (RIG-I) and IFN regulatory factor 1 (IRF-1) [60].

Vitamin B may control the production of chemokines and cytokines and control how immune cells communicate in pathogenic pathways. Additionally, since probiotics like bifidobacteria and lactobacilli have been shown to influence immune responses and guard against infections, vitamin B may be beneficial for immunity since it regulates colonic immune function and helps the intestinal barrier function [14].

Vitamin C and vitamin D are immunomodulating substances that have been used for decades to treat a variety of diseases. Vitamins D and C inhibit the release of proinflammatory cytokines in some immune system components and promote the growth of other immune cells, allowing the host to combat infection effectively while not depleting its immunological and energy reserves [61]. Vitamin C is an antioxidant that has a significant impact on both innate and adaptive immunological responses, as well as microbial metabolism [12]. Vitamin C treatment protects rat liver function against cyclosporine-A-induced damage by lowering ALT, AST, LDH, and ALP levels while increasing total protein and albumin levels [62].

As a strong antioxidant, vitamin E is critical in regulating and sustaining immune system activity [63]. Vitamin E is a free radical scavenger that decreases oxidative stress and protects cells against extremely energetic and damaging free radicals with unshared electrons [64]. When unshared electrons react quickly with oxygen, they generate reactive oxygen species (ROS). Vitamin E is key in immunity in addition to its antioxidant and anti-inflammatory activities [65].

Folate is a B vitamin that is required for DNA and protein synthesis, as well as the adaptive immune response [66]. The deleterious effects of folate deficiency on immune function are most likely mediated by anomalies in DNA and RNA synthesis or methyl metabolism, both of which are heavily influenced by folate availability [12]**.**

Zinc is an important metal that plays a role in a range of biological processes as a cofactor, signalling molecule, and structural element. It possesses antiviral and antioxidant properties and modulates inflammatory activity [67]. Zn deficiency promotes oxidative stress, pro-inflammatory TNF-α, and vascular cell adhesion molecule (VCAM)-1 expression in rats, according to studies [68].

The star anise essential oil was employed to hide the unpleasant taste of vitamins and minerals in the current study. Aside from its pleasant aroma, star anise essential oil is a rich source of the shikimic acid molecule, which is utilized by pharmaceutical companies to create Tamiflu, an anti-influenza drug. *Trans*-anethole, the major fragrance component of star anise oil (as observed from our results Table 3 and Fig. 1), was also found to have anti-inflammatory properties, lowering TARC, MDC, and the cytokines IL-6 and IL-1β [69]. As noticed from the results presented in Table 2, star anise oil has antioxidant effects, which are thought to be linked to the presence of *trans*-anethole, namely its double bonds in the molecules [70]. As a result, star anise oil in the VFnPs may play an important role in preventing cyclosporine-A side effects.

### 3.4 Physiochemical properties in ice cream mixes

- **The specific gravity, weight per gallon**, of ice cream mixes are illustrated in Table 7. The mean values of specific gravity were 1.052, 1.078 and 1.095 g/cm^3^ for the control, T1, and T2, respectively. From the obtained results, it could be observed that the specific gravity increased with the increasу in the percentage of encapsulated star anise into milk powder. On the other hand, the weight per gallon (Kg) of ice cream mixes were closely related to the specific gravity and were recorded 3.996, 4.233 and 4.254 for the control, T1, and T2, respectively [71].

**Acidity** pH **values**: the acidity of ice cream mixes was 0.73, 0.36, and 0.38, and the pH values take an opposite trend of the acidity. These data indicated that the acidity of mixes was nearly the same and was affected by high total solids in all treatments [72].

**Ash contents %**: the ash percentage of ice cream mixes were 0.79, 0. 82 and 0.85 % for the control, T1, and T2, respectively. The obtained results indicated that the ash contents slightly increased with increasing the percentage of milk powder with encapsulated star anise.

### 3.5 Physiochemical properties in the resultant ice cream

**The specific gravity, weight per gallon**, of the resultant ice cream are shown in Table 8. The mean values of specific gravity were 0.891, 0.844 and 0.823 g/cm^3^ for the control, T1, and T2, respectively. The weight per gallon (Kg) of the resultant ice cream were closely related to the specific gravity and were recorded as 3.45, 3.27 and 3.18 for the control, T1, and T2, respectively [73].

**Table 7:** Physiochemical properties of ice cream mixes.

Parameters	Control	T1	T2
**Specific gravity (g/cm**^**3**^**)**	1.052	1.078	1.095
**Weight per gallon (Kg)**	3.996	4.233	4.254
**Acidity**	0.37	0.36	0.38
pH **values**	5.95	6.11	5.98
**Ash**	0.79	0.82	0.85

**Table 8:** Physiochemical and sensory properties of resultant ice cream.

Parameters	Control	T1	T2
Specific gravity	0.891	0.844	0.823
Weight per gallon	3.45	3.27	3.18
Overrun	48.58	50.90	54 .67
Melting rate% after 30 min	94	89	82
Total score of sensory evaluation (100 %)	91	88	89

**Overrun%**: the overrun was 48.58, 50.90 and 54.67 % for the control, T1, and T2, respectively. The results showed that the increase in the overrun was due to the increase of milk powder and total solids in the mixes, therefore T2 had the highest overrun percentage [74].

**Melting rate %**: the melting rate of the resultant ice cream was 94, 89 and 82 % for the control, T1, and T2, respectively. It could be seen that the high melting rate was recorded in control ice cream, but with increasing the milk powder into T1 and T2 leads to high melting resistance compared with control one. The milk powder increased the total solids in mixes and enhancing the consistency and meting rate ability [74,75].

### 3.6 Sensory evaluation of functional ice cream

The mean of sensory evaluation scores for ice cream is illustrated in Table 8. The results showed that the control had the highest scores, followed by the treatment 2, which had 0.66 % of encapsulated star anise oil. Finally, functional ice cream fortified with encapsulated star anise oil and encapsulated vitamins had higher quality and acceptability, which could be due to the combination of the ingredients of mixes and star anise oil. Increasing the ratio of encapsulated star anise and vitamins has slight effect on the sensory evaluation of the ice cream. The improved body and texture of ice cream may be attributed to the increase of total solids in the mixes. The findings are consistent with those reported by **Abdel-Haleem and Awad** [71]**.**

This study's main limitation was that it was unable to perform the experiment on volunteers. The pre-study on animals was necessary to gather sufficient information regarding the likely biological effects and reasonable safety of these newly formulated flavoured nanophytosomes and ice cream, even though mineral and vitamin mixtures are classified as dietary supplements. Thus, more research examining the impact of these flavoured nanophytosomes and ice cream fortified with it is needed.

## 4 Conclusion

The results revealed no statistically significant differences between samples containing vitamins-loaded flavoured nanophytosomes and control ones. It can be concluded that vitamins-loaded flavoured nanophytosomes may be offered as an efficient natural food preservative. Flavoured nanophytosomes containing vitamins were developed for ice-cream to overcome the major problems with flavoured vitamins and improve the immune system of the human body. Along with its function as an immune-enhancing agent, vitamins-loaded flavoured nanophytosomes may have a potential as a hepatoprotective due to its antioxidant and anti-inflammatory actions. After full characterization, vitamins-loaded flavoured nanophytosomes were used in ice cream.

## Data availability statement

The data that support the findings of this study are available within the article.

## CRediT authorship contribution statement

**Manal M. Ramadan:** , Supervision, Project administration, Investigation, Conceptualization. **Rasha S. Mohamed:** , Visualization, Validation, Methodology, Investigation, Formal analysis. **Amal G. Hussien:** , Writing – original draft, Validation, Methodology, Conceptualization. **Ola A.M. Mohawed:** , Writing – original draft, Methodology. **Ahmed M. Mabrouk:** Writing – original draft, Methodology. **Abeer E. Mahmoud:** , Writing – original draft, Methodology. **Kadry Z. Ghanem:** , Validation, Formal analysis. **Tamer M. El-Messery:** Writing – review & editing, Visualization.

## Declaration of competing interest

The authors declare that they have no known competing financial interests or personal relationships that could have appeared to influence the work reported in this paper.
